# Author’s Reply: HFE Gene Mutations (C282Y and H63D) in a Group of Patients With Cryptogenic Cirrhosis

**DOI:** 10.5812/kowsar.1735143X.821

**Published:** 2012-01-20

**Authors:** Bita Geramizadeh

**Affiliations:** 1 Department of Pathology, Shiraz University of Medical Science, Shiraz, IR Iran

**Keywords:** Patients, Genes, Iran

Dear Editor,

I would like to thank Dr Sendi and Mohseni for their interest in our study.In this letter the authors have summarized the contributory effects of hereditary hemochromatosis (HH) in liver diseases in different populations [1]. I completely agree with them concerning the underlying causes of cryptogenic cirrhosis which in countries such as Iran is most commonly nonalcoholic steatohepatitis (NASH). In our study we tried to demonstrate that the epidemiology of the HH and HFE gene mutation in Iran is completely different from that found in the West, and also to emphasize the infrequency of the C282Y mutation in Iran [2]. In recent publications from countries such as India, similar results were found and all the data were in favor of a minor contributory role of HH in the development of cirrhosis in these countries which is different from in the West [3]. Finally I want to emphasize the effect that the presence of the HFE gene mutation has on the fibrogenesis of other liver diseases such as alcoholic or viral hepatitis [4] and this should be investigated in future studies in Iran.
